# Regenerating Salivary Glands in the Microenvironment of Induced Pluripotent Stem Cells

**DOI:** 10.1155/2015/293570

**Published:** 2015-06-22

**Authors:** Hitomi Ono, Aya Obana, Yu Usami, Manabu Sakai, Kanji Nohara, Hiroshi Egusa, Takayoshi Sakai

**Affiliations:** ^1^Department of Oral-Facial Disorders, Osaka University Graduate School of Dentistry, Suita, Osaka 565-0871, Japan; ^2^Division of Orthopedic Surgery, The Children's Hospital of Philadelphia Research Institute, Philadelphia, PA 19104, USA; ^3^Clinical Laboratory, Osaka University Dental Hospital, Suita, Osaka 565-0871, Japan; ^4^Division of Molecular and Regenerative Prosthodontics, Graduate School of Dentistry, Tohoku University, Sendai, Miyagi 980-8575, Japan

## Abstract

This report describes our initial attempt to regenerate salivary glands using induced pluripotent stem (iPS) cells *in vivo* and *in vitro*. Glandular tissues that were similar to the adult submandibular glands (SMGs) and sublingual glands could be partially produced by the transplantation of iPS cells into mouse salivary glands. However, the tumorigenicity of iPS cells has not been resolved yet. It is well known that stem cells affect their microenvironment, known as a stem cell niche. We focused on the niche and the interaction between iPS cells and salivary gland cells in our study on salivary gland regeneration. Coculture of embryonic SMG cells and iPS cells have better-developed epithelial structures and fewer undifferentiated specific markers than monoculture of embryonic SMG cells *in vitro*. These results suggest that iPS cells have a potential ability to accelerate differentiation for salivary gland development and regeneration.

## 1. Introduction

Salivary glands have important functions in maintaining oral health [1]. Hypofunction of the salivary glands can cause various life-disrupting side effects, such as dental caries, swallowing difficulties, loss of taste, and oral candidiasis. Irradiation therapy and Sjögren's syndrome can cause salivary gland hypofunction and xerostomia. No satisfactory therapy has been established to treat salivary hypofunction [1–3].

Induced pluripotent stem (iPS) cells can be generated from fully differentiated nonpluripotent cells and possess pluripotency similar to that of embryonic stem (ES) cells [4]. iPS cells could be a powerful tool in regenerative medicine, but their potential tumorigenicity is a significant challenge for clinical use [5, 6].

On the other hand, ES cells and other stem cells have a microenvironment that is self-renewing and have multilineage developmental potential.* In vivo*, these properties are not autonomous to stem cells, and recent evidence points to a level of external control from the microenvironment that defines the stem cell niche. The stem cell niche may represent a significant entry point for therapeutic modulation of stem cell behavior [7, 8]. Although it is known that the microenvironment is derived from iPS cells, the niche of iPS cells has not been well studied [9, 10].

In this study, we tried to regenerate salivary glands using both embryonic salivary glands and iPS cells, and we identified putative functions of the iPS cell niche. Many researchers have tried to regenerate organs, such as salivary glands, kidneys, and lungs [11–13]. A recent study described a therapy using stem/progenitor cell transplantation after radiation to restore long-term saliva production [3]. It is currently proposed that stem/progenitor cells reside in the ducts of salivary glands [14]. There is a possibility that the stem cell niche affects salivary gland development and regeneration. These analyses may provide new concepts for the functional regeneration of salivary glands using tissue engineering.

## 2. Methods

### 2.1. Animals

All animal experiments were performed in strict accordance with the guidelines of the Animal Care and Use Committee of the Osaka University Graduate School of Dentistry, Osaka, Japan. The protocol was approved by the Committee on the Ethics of Animal Experiments of Osaka University Graduate School of Dentistry (permit number: 25-004-0). All surgeries were performed under sodium pentobarbital anesthesia, and all efforts were made to minimize suffering. SMGs were dissected from embryonic and postnatal ICR mice (Japan SLC, Inc., Hamamatsu, Japan). For regenerating cultures of salivary glands, three mice in embryonic day 13.5 (E13.5) were used. For the analysis of developmental process, three mice in E13.5 to E17.5 and three mice in postnatal eight weeks were used. For teratoma formation assays, three immunodeficient mice (C.B-17 SCID, Clea Japan, Tokyo, Japan) that are eight-week-old were used. Each experiment was repeated at least three times, with data showing a representative experiment.

### 2.2. Cell Culture

The mouse green fluorescent protein- (GFP-) iPS cell line (APS0006: iPS-Stm-FB/gfp-99-3), derived from stomach cells, was provided by the RIKEN BRC through the National Bio-Resource Project of the MEXT, Japan. The iPS cells express GFP by the CAG promoter. The cells were disseminated in a 6-well dish with gelatin solution and cultured with ESGRO complete plus serum-free clonal grade medium (Millipore, Billerica, MA, USA). The cells were washed once with D-PBS (Millipore, Billerica, MA, USA) and dissociated with Accutase cell detachment (Millipore, Billerica, MA, USA).

### 2.3. Teratoma Formation Assay

To analyze the multipotency of GFP-iPS cells, 2.0 × 10^5^ GFP-iPS cells were collected as one pellet in cold Dulbecco's Modified Eagle's Medium/F12 (DMEM/F12) (Invitrogen) and transplanted into the SMG of anesthetized SCID mouse. The mice were thereafter housed with free access to water and food under specific pathogen-free conditions. The teratomas were excised after 4 weeks, fixed in 4% PFA, embedded in paraffin, and sectioned at 3 *µ*m. Teratoma formation assays were repeated at least three times using three mice per group, with data showing a representative experiment. The histology of the formed teratomas was analyzed using hematoxylin and eosin (H&E) staining.

### 2.4. Regenerating Salivary Gland Culture

SMGs were treated with trypLE (Life technologies, Carlsbad, CA, USA), and the dissociated cells were seeded on 20 *µ*L matrigel (BD Biosciences, Franklin Lakes, NJ, USA) coated U-bottom plates (Thermo, Waltham, MA, USA) to yield 2.0 × 10^5^ cells per well. Then, the cells were cultured with 250 *µ*L serum-free DMEM/F12 medium for 96 h, as previously described. Monoculture of SMG cells is defined as SG, and coculture of SMG cells and GFP-iPS cells is defined as iSG. 5% iSG consists of 5% iPS cells and 95% SMG cells. 20% iSG consists of 20% iPS cells and 80% SMG cells.

### 2.5. Immunofluorescence

Paraffin-embedded tissues of teratomas and regenerated salivary glands were evaluated. Tissue sections were deparaffinized, and antigen retrieval was performed by autoclave heating (instant antigen retrieval H buffer, 121°C for 5 min). The slides were washed in phosphate-buffered saline (PBS). The samples were first incubated with M.O.M. Mouse Ig Blocking Reagent (Vector Laboratories, Inc., Burlingame, CA, USA) and then with primary antibodies in diluent (1x PBS, containing 8% protein concentrate; M.O.M Kit, Vector Laboratories, Inc.) overnight at 4°C. Specific antibodies used were anti-*α*-Amylase (dilution 1 : 100; Sigma-Aldrich, St. Louis, MO, USA), anti-parotid secretory protein (PSP) (dilution 1 : 100; Everest Biotech, Oxfordshire, UK), anti-E-cadherin (dilution 1 : 100; BD Biosciences, Franklin Lakes, NJ, USA), anti-Green Fluorescent Protein (GFP) (dilution 1 : 100; MBL, Nagoya, Japan), anti-SRY (sex determining region Y) box 2 (Sox2) (dilution 1 : 100; Santa Cruz, Dallas, TX, USA), and anti-Aquaporin 5 (AQP5) (dilution 1 : 100; Alomone, Har Hotzvim, Israel). After washing with PBS, the tissues were incubated with Cy2-labelled donkey anti-Goat and Cy3-labelled donkey anti-mouse and Cy5-labelled donkey anti-rabbit or anti-rat IgG for 2 h at room temperature (dilution 1 : 100; Life Technologies, Carlsbad, CA, USA) in diluent (5% donkey serum, containing 8% protein concentrate). Immunostaining was repeated at least three times.

### 2.6. Extraction of the SMG Cells from Regenerated Salivary Glands by Cell Sorting

SG and iSG were treated with trypLE (Life technologies, Carlsbad, CA, USA), and SMG cells in each SG and iSG were dissociated to single cells. SMG cells were separated from GFP-iPS cells using a fluorescent-activated cell sorter (FACSAria, BD Biosciences, Franklin Lakes, NJ, USA). GFP-iPS cells were separated from the GFP fraction by fluorescent-activated cell sorting. SMG cells from SG and from iSG were analyzed by quantitative RT-PCR (qPCR) and by western blotting.

### 2.7. Quantitative Real-Time Reverse Transcription-Polymerase Chain Reaction

SMG cells were separated from iPS cells by cell sorting. Total RNA was isolated from both embryonic and postnatal tissues using TRIzol Reagent (Life Technologies, Carlsbad, CA, USA) according to the manufacturer's instructions and was treated with DNase I (Roche Applied Science, Penzberg, Upper Bavaria, Germany) to avoid genomic DNA contamination. For cDNA synthesis, reverse transcription was performed using the Prime Script RT Reagent Kit (Takara Bio Inc., Otsu, Shiga, Japan). Quantification of PCR products was performed using the MyiQ Single-Color Real-Time PCR Detection System (Bio-Rad Laboratories, Inc., Hercules, CA, USA) with iQ SYBR Green Supermix (Bio-Rad Laboratories, Inc., Hercules, CA, USA). The amplification program comprised 40 cycles of denaturation at 95°C for 5 s, annealing at 55°C for 20 s, and extension at 72°C for 20 s. Quantitative real-time reverse transcription-polymerase chain reaction (qPCR) results for each sample were normalized by glyceraldehyde-3-phosphate dehydrogenase (*Gapdh*). The results were expressed as normalized ratios, and experiments were repeated three times. The primer sequences used were as follows:

 
*Sox2*: 5′-GGGAAATGGGAGGGGTGCAAAAGAGG-3′.
 5′-TTGCGTGAGTGTGGATGGGATTGGTG-3′.
 
*c-Myc*: 5′-CAGAGGAGGAACGAGCTGAAGCGC-3′.
 5′-TTATGCACCAGAGTTTCGAAGCTGTTCG-3′.
 
*Klf4*: 5′-CACCATGGACCCGGGCGTGGCTGCCAGAAA-3′.
 5′-TTAGGCTGTTCTTTTCCGGGGCCACGA-3′.
 
*Aqp5*: 5′-TGGAGCAGGCATCCTGTACT-3′.
 5′-CGTGGAGGAGAAGATGCAGA-3′.
 
*Amy*: 5′-GGATGGAGAAAAGATGTCCTAC-3′.
 5′-CATCACCCGTGTGAAACC-3′.
 
*M3r*: 5′-TCGGTAGAGCGGACTGGACA-3′.
 5′-TCCACTGAGCAAGTCAGAAGTGAAG-3′.
 
*Gapdh*: 5′-CCATCACCATCTTCCAGGAG-3′.
 5′-GCATGGACTGTGGTCATGAG-3′.

### 2.8. Statistical Analysis

Statistical analysis was performed as indicated in the figure legends and text. One-way analysis of the *t*-test was performed to determine the statistical difference between the means of each treatment for experiments in which multiple treatments were compared.

## 3. Results

### 3.1. Teratoma Formation in SMG after Transplantation of Mouse GFP-iPS Cells

**Figure 1:**
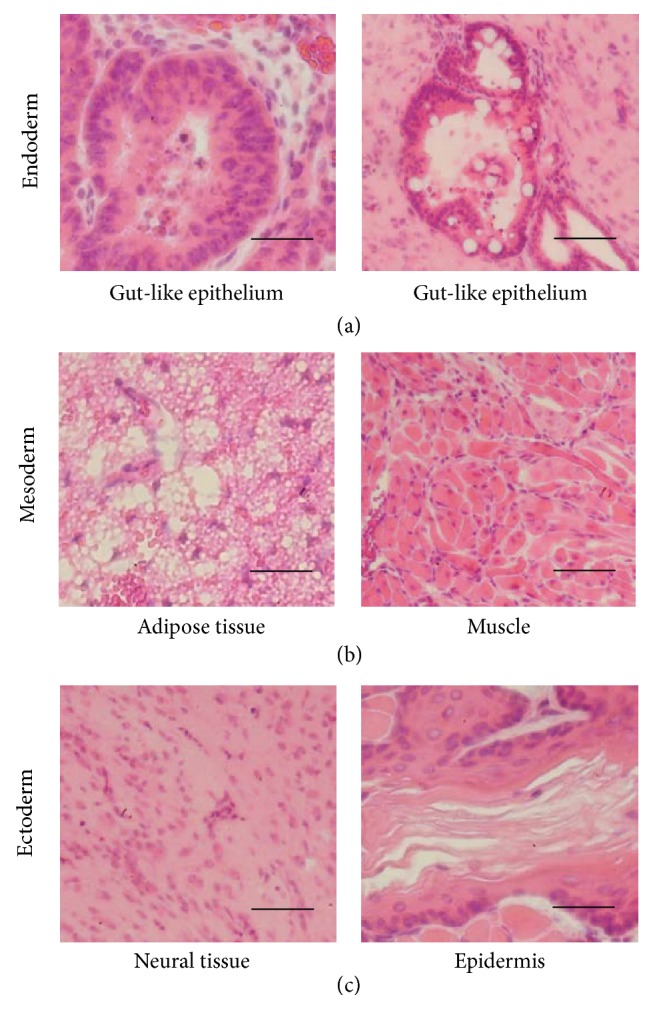
Histological analysis of teratoma formation after transplantation of GFP-iPS cells. Images of sections of teratomas formed after transplantation of GFP-iPS cells into salivary glands of SCID mice. A teratoma formed from transplantation of 5.0 × 10^5^ GFP-iPS cells. H&E stained sections of a teratoma showing derivatives of all three germ layers, including gut-like epithelium tissues ((a) endoderm), adipose tissues and muscles ((b) mesoderm), and neural tissues and keratin-containing epidermal tissues ((c) ectoderm). Scale bars, 50 *µ*m.

**Figure 2:**
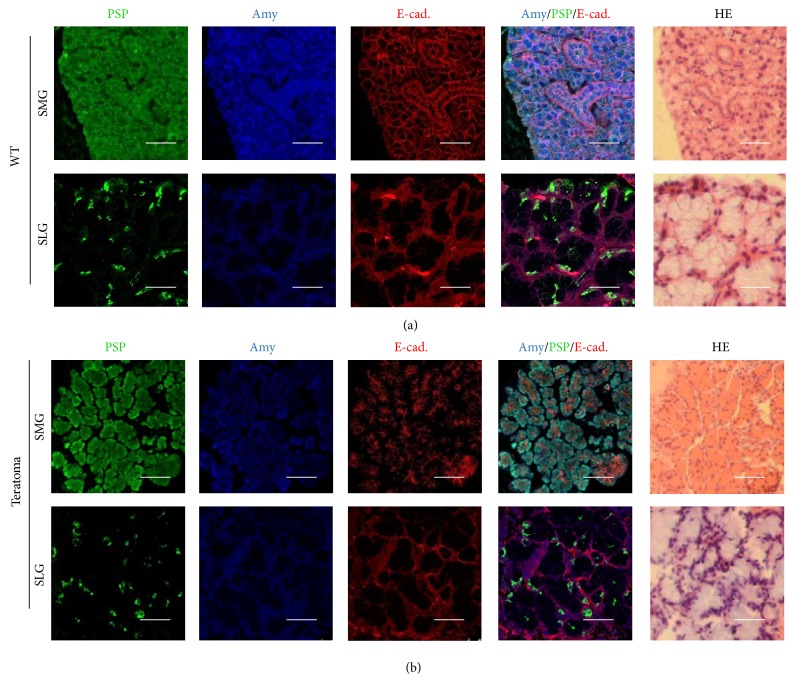
Histological analysis of salivary gland-like tissue in teratomas formed after transplantation of GFP-iPS cells. Immunofluorescence and H&E staining of sections showed submandibular glands (SMGs) and sublingual glands (SLGs) of adult mouse. PSP (green), Amy (blue), and E-cadherin (red) (a). Scale bars, 25 *µ*m. Immunofluorescence and H&E staining of sections of GFP-iPS-grafted teratoma revealed salivary gland-like tissue (b). Scale bars, 25 *µ*m.

The pluripotency of GFP-iPS cells was validated by teratoma formation in the SMG of SCID mice. Four weeks after implantation, histological analysis demonstrated that the formed teratomas were derived from all three primary germ layers. Gut-like epithelium (endoderm), adipose tissues (mesoderm), muscles (mesoderm), neural tissues (ectoderm), and epidermis (ectoderm) were all identified histologically in the GFP-iPS-derived teratomas (Figure 1). The teratomas contained different types of salivary gland-like tissues. By immunohistochemical analysis, one was similar to the SMG, and the other was similar to the pattern of the SLG in adult mice (Figure 2).

### 3.2. Localization of iPS Cells in Regenerated SMGs (SG and iSG)

**Figure 3:**
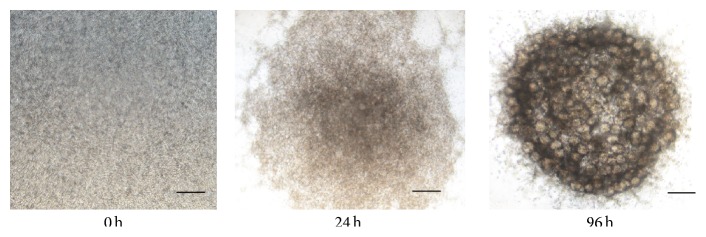
Aggregation of SMG cells in SG. Phase-contrast image of regenerated SMG in organ culture for 0, 24, and 96 h. The regenerated SMG contained many acinar-like structures. Scale bar, 0.5 mm.

**Figure 4:**
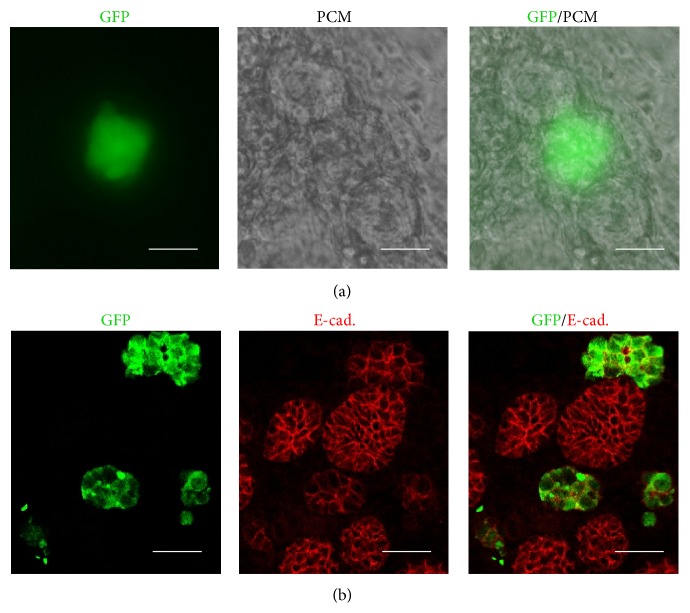
Aggregation of SMG cells in iSG. SMG cells and GFP-iPS cells were cocultured with DMEM/F12 for 96 h. Localization of GFP-iPS cells in iSG. iSG consists of 20% iPS cells and 80% SMG cells. Phase-contrast image and immunological stained image GFP-iPS (green) and pericentriolar material (PCM) (a). Scale bar, 25 *µ*m. Immunofluorescence of paraffin-embedded tissue of regenerated salivary glands. GFP-iPS (green) and E-cadherin (red) (b). Scale bar, 25 *µ*m.

In SMG monoculture, the epithelial cells aggregated and formed salivary gland-like tissue (SG) after 96 h (Figure 3). Coculture of SMG cells and GFP-iPS cells (iSG) also formed many acinar-like structures similar to the SG. In immunohistochemical analysis, GFP-iPS cells did not uniformly mix with SMG cells (Figure 4(a)). GFP-iPS cells existed around acinar-like epithelial cells (Figure 4(b)).

### 3.3. Morphological Analysis of Regenerated Salivary Glands (SG, 5% iSG, and 20% iSG)

**Figure 5:**
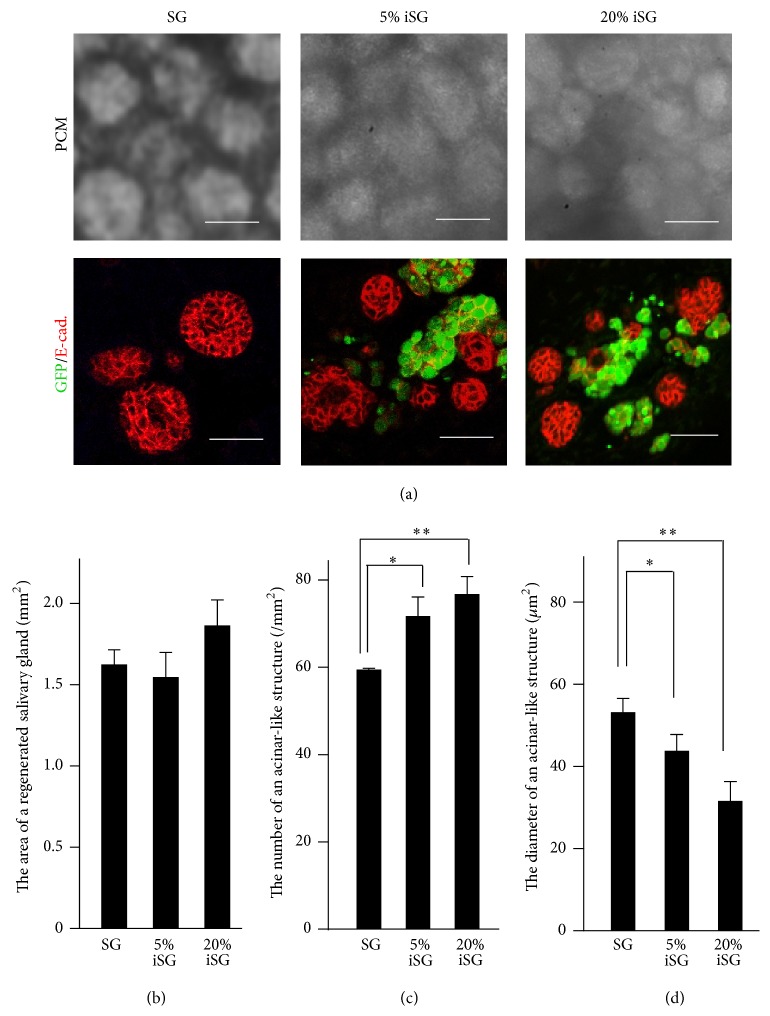
Morphological analysis of regenerated SMGs (SG, 5% iSG, and 20% iSG). PCM and immunostained images indicated a reduction in size and an increase in the number of acinar-like structures in regenerated salivary glands (a). Size of whole regenerated salivary glands (*n* = 3) (b). Number of acinar-like structures in regenerated salivary glands (*n* = 3) (c). Size of acinar-like structures in regenerated salivary glands (*n* = 24) (d). ^*∗*^ *p* < 0.05; ^*∗∗*^ *p* < 0.01 versus control.

To investigate the effect of GFP-iPS cells on SMG cells, the morphology of SG and iSG was analyzed (Figure 5(a)). The size of regenerated salivary glands was statistically evaluated. There were no significant size differences between SG, 5% iSG, and 20% iSG (Figure 5(b)). The number of acinar-like aggregations in 5% iSG and 20% iSG was more than that in SG (Figure 5(c)). The size of acinar-like structures was significantly reduced in 5% iSG and 20% iSG in comparison with that of SG (Figure 5(d)).

### 3.4. Expression of Differentiation Markers in Regenerated Salivary Glands (SG and iSG)

**Figure 6:**
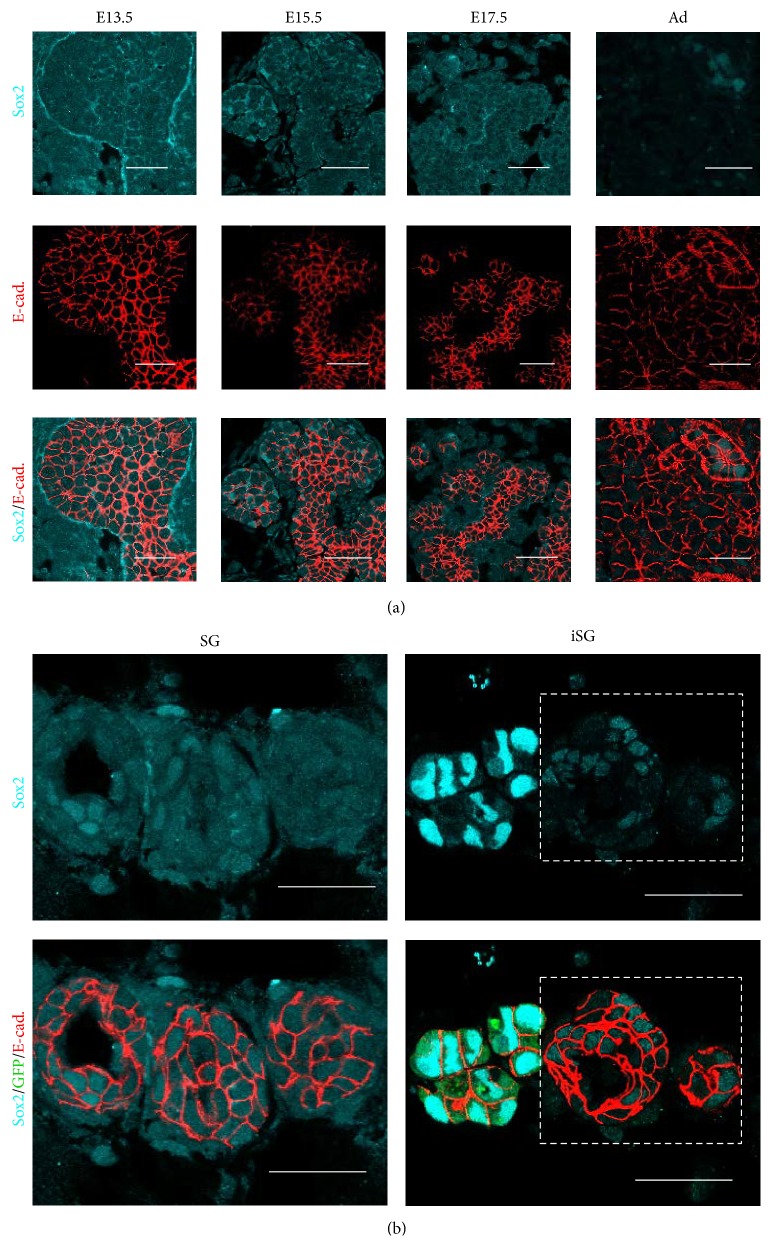
Distribution of Sox2 in developing SMG and in regenerated SMGs (SG and iSG). Sox2 is expressed in the cytoplasm of both epithelial cells and mesenchymal cells in E13.5, E15.5, and E17.5 developing SMGs and is gradually decreased in later embryonic stages. Sox2 is expressed in the nucleus of epithelial cells in both postnatal and adult SMGs. Sox2 (cyan) and E-cadherin (red) (a). Scale bar, 25 *µ*m. Sox2 expression in the cytoplasm of both epithelial cells and mesenchymal cells in iSG (20% iSG) is less than that in SG. The nucleus of GFP-iPS cells highly expressed Sox2 as a positive control. Sox2 (cyan), GFP (green), and E-cadherin (red) (b). Scale bar, 25 *µ*m.

To evaluate the differentiation of SMG cells, embryonic stem cell markers and salivary gland markers were used for qPCR and for immunohistological analysis.* Sox2* is known as a transcription factor involved in regulating the self-renewal of embryonic stem cells. The development of the SMG is an ideal model to study the differential stage of regenerated salivary glands. Sox2 was expressed both in the epithelium and mesenchyme of E13.5 mouse SMGs and in the epithelium of E15.5 to E17.5. Sox2 was gradually decreased from E17.5 to the adult stage and was expressed in the nucleus of ductal epithelium but not in the cytoplasm (Figure 6(a)). The expression of Sox2 was induced in the cytoplasm of SG, but decreased in the cytoplasm of iSG (Figure 6(b)).

**Figure 7:**
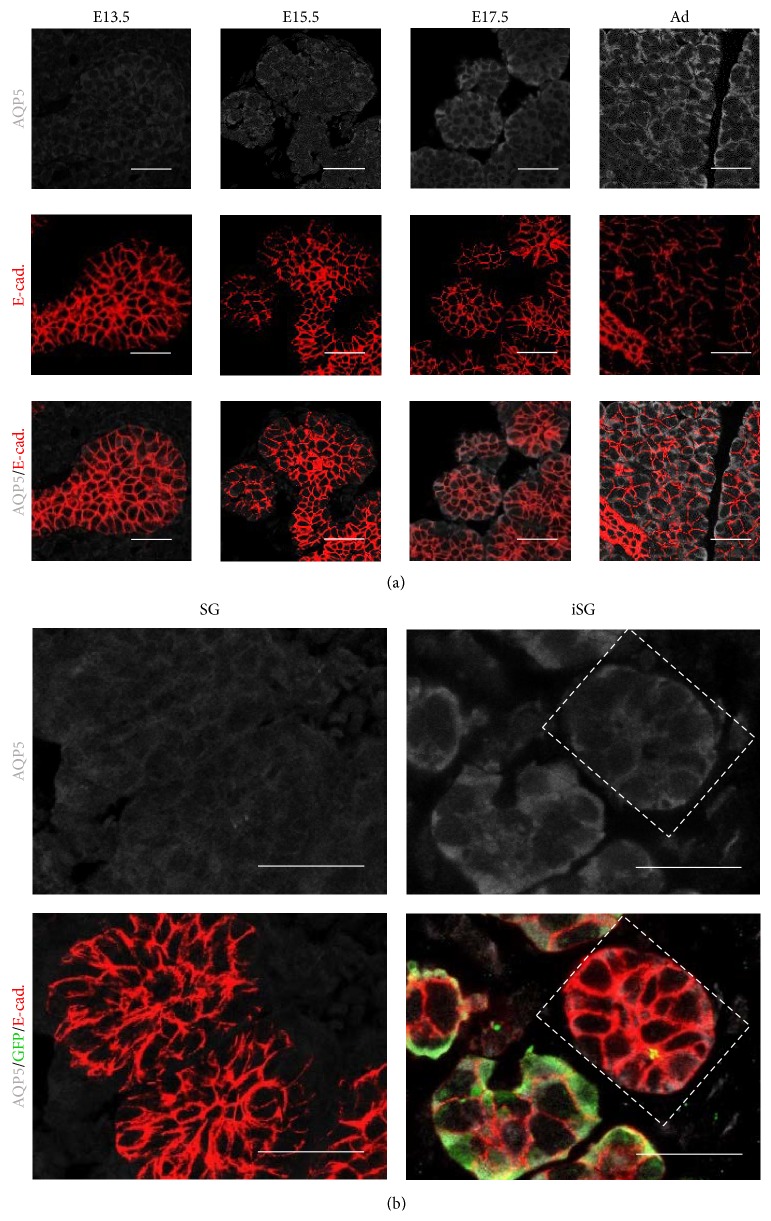
Distribution of AQP5 in developing SMG and regenerated SMGs (SG and iSG). AQP5 is gradually increased in the epithelial cells of SMGs in E13.5, E15.5, E17.5, and adult. AQP5 is expressed in the cell membrane of epithelial cells in iSG (20% iSG) more than that in SG. AQP5 (white) and E-cadherin (red) (a). Scale bar, 25 *µ*m. Distribution on AQP5 in regenerated SMG (b). AQP5 (white), GFP (green), and E-cadherin (red). Scale bar, 25 *µ*m.

AQP5 is known as one of the salivary gland markers for fluid secretion. AQP5 was expressed at a low level in the terminal bud and in the epithelial stalk of E13.5 and E15.5 SMGs. AQP5 was increased in the organized proacinar cells of SMGs in E17.5 to adult (Figure 7(a)). SMG cells in SG expressed AQP5 at the same level as E13.5 SMG. However, SMG cells in iSG highly expressed AQP5 at the same level as that in E17.5 SMG (Figure 7(b)).

**Figure 8:**
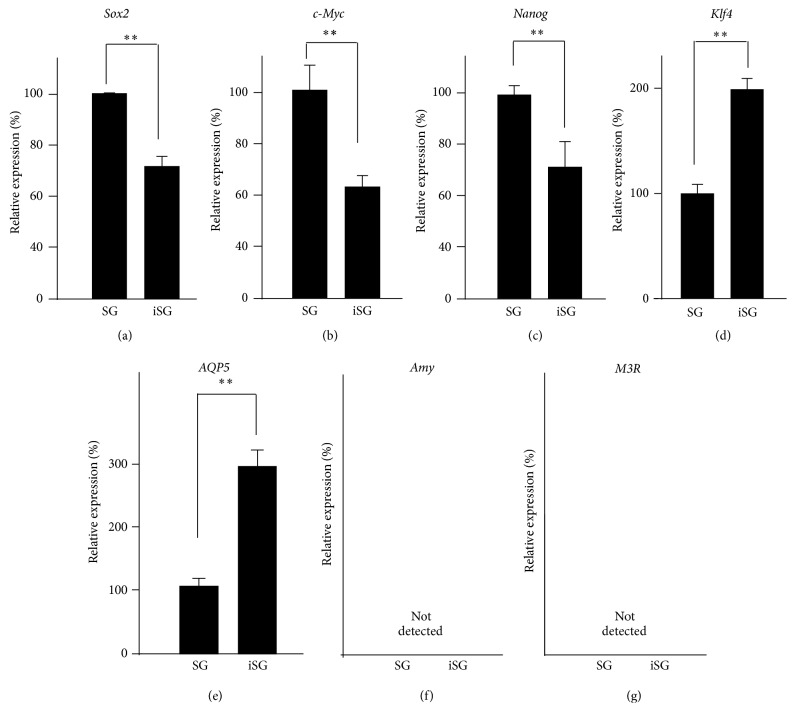
Gene expression of salivary gland cells in regenerated SMGs between SG and iSG. Gene expression of* Sox2* (a),* c-Myc* (b), and* Nanog* (c) was decreased in iSG, and gene expression of* Klf4* (d) was increased in iSG.* Aqp5* (e) gene expression was increased in iSG, but* Amy* (f) and* M3r* (g) gene expression were not detected by qPCR, similar to early embryonic SMG cells. ^*∗∗*^ *p* < 0.01 versus control.

To analyze specific gene expression, SMG cells from SG and iSG were isolated by a cell sorter. The gene expression of markers related to embryonic stem cell maintenance (*Sox2, Nanog, c-Myc, and Klf4*) and submandibular markers (*Aqp5*,* Amy*, and* M3r*) was investigated by qPCR. The gene expression of* Sox2, c-Myc*, and* Nanog* was decreased in iSG compared with that in SG. However,* Klf4* was increased in iSG (Figure 8(a)–8(d)).* Aqp5* gene expression was higher in iSG than that in SG (Figure 8(e)). The expression of* Amy* and* M3r* was not detected by qPCR (Figures 8(f) and 8(g)).

## 4. Discussion

Salivary glands have a potential ability to recover their function over time [15]. Several studies showed that stem/progenitor cells in salivary glands restored salivation [16–18]. Various kinds of cells have been used for transplantation in regenerative medicine. Many studies showed that the stem cell itself differentiates in the part of the organ where the function was restored. Recent studies revealed that various kinds of cytokines secreted by stem cells are related to the mechanism of regeneration. The transplantation of bone marrow stromal cells for spinal cord injury produced extensive outgrowth of regenerating axons associated with Schwann cells through the extracellular matrices [8, 19]. The restoration of morphology and function was induced by the combination of several factors, such as the paracrine effect, cell transdifferentiation, and vasculogenesis by hematopoietic and/or mesenchymal cells derived from bone marrow [20].

Many studies have tried to use iPS cells as a source of the transplant for treatment [21, 22]. However, the function of the microenvironment that iPS cells produce has not been investigated yet. In this study, we examined the differentiation of salivary gland cells using iPS cells in a microenvironment.

At first, to confirm the pluripotency of iPS cells, GFP-iPS cells were transplanted into SCID mice. Histological analysis of the produced teratoma demonstrated all three primary germ layers. It suggested that GFP-iPS cells had pluripotency for differentiation. Furthermore, teratomas contained two kinds of glandular tissues which were similar to both the SMG and the SLG, compared with the pattern of the salivary glands in adult mice [23]. GFP-iPS cells have a potential ability to regenerate SMG and SLG cells. However, only few parts of the tissues differentiated from salivary glands. The potential tumorigenicity of iPS cells has to be considered prior to clinical application.

The embryonic SMG is a remarkably adaptable tissue, and dissociated SMG cells can self-assemble in serum-free medium [24]. The cell aggregation assay is a useful method for studying mechanisms of tissue assembly, as the structure of the regenerated glands is similar to salivary glands [25, 26]. Embryonic SMG cells and GFP-iPS cells were cocultured to identify the roles of the microenvironment around the iPS cells. Regenerated salivary glands (SG and iSG) formed many acinar-like structures similar to embryonic salivary glands for 96 h* in vitro* [27]. It is very difficult for our conventional organ culture without serum and other factors to grow salivary gland* in vitro* for longer time such as several weeks. So salivary gland cells needed to be transplanted to mouse [28, 29].

**Figure 9:**
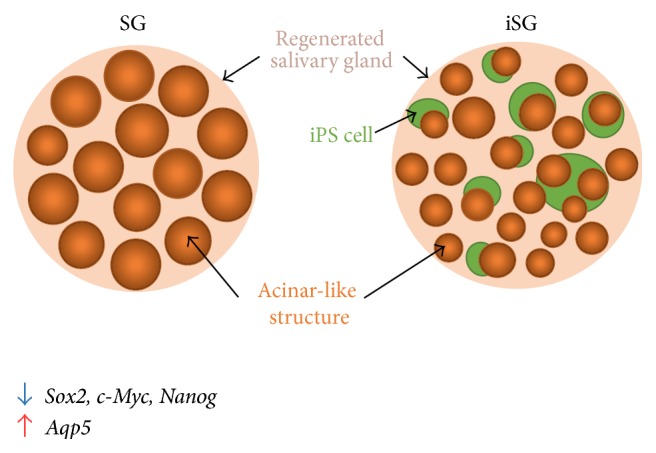
Effect of iPS cells on aggregation of SMG cells. iPS cells (green balls) reduced the size of the epithelial tissue, but acinar-like structure (orange balls) increased their number. iPS cells cannot mix completely with SMG cells and, instead, surround the epithelium of SMG. Stem cell markers, such as* Sox2*,* c-Myc*, and* Nanog*, are decreased, but* Aqp5* is increased in iSG.

Because the water channel protein AQP5 is found in the lumen of the acinar-like structures, regenerated salivary glands may have an ability to secrete saliva [30, 31]. Morphological analysis of the regenerated salivary glands indicated that the difference between SG and iSG was not statistically significant. However, iSG had a larger number of small acinar-like structures, more than that in SG (Figure 9). Developing salivary glands in epithelial tissue increases the surface area by branching morphogenesis and increases the ability of saliva secretion [32–34]. Coculture of embryonic SMG cells and iPS cells had more developed epithelial structures than that in monoculture of embryonic SMG cells.

Previous studies show the gene expression of stem cell markers (*Sox2, c-Myc, Nanog, and Klf4*) in E13, E15, and adult (Ad) SMGs.* Sox2, c-Myc*, and* Nanog* decreased during SMG development, but* Klf4* increased [16]. The submandibular marker AQP5 belongs to a family of water channel proteins that allow water to pass through the plasma membrane by osmosis. E14 SMG expressed AQP5 as a first indication [31]. Our results showed that the gene expressions of* Sox2, c-Myc*, and* Nanog* were decreased in iSG and that the gene expression of* Klf4* and* Aqp5* was increased in iSG. Immunohistochemical analysis also showed that the undifferentiated marker of salivary gland cells decreased in iSG compared with that in SG. It was suggested that iPS cells induce the characteristic differentiation resulting in the morphological changes observed in salivary gland cell formation. These results indicate that iPS cells have a potential ability to accelerate differentiation of salivary gland development and regeneration. Regenerated salivary glands produced from E13.5 SMG cells were cultured for four days.* Amy* and* M3r* are usually expressed after birth.* Amy* and* M3r* gene expression also occurred in the regenerated salivary gland cells.

Various approaches would be necessary to organize the complex tissues, such as the salivary gland, kidney, and lung. Our finding that coculture of salivary glands cells with iPS cells formed differentiated salivary glands is significant, and future elucidation of the mechanism could lead to viable regeneration therapy of functional organs using iPS cells. Our study provides new insights for future research into the regeneration of organs, such as salivary glands.
